# Predictors of Good Long-Term Renal Outcomes in Lupus Nephritis: Results from a Single Lupus Cohort

**DOI:** 10.1155/2017/5312960

**Published:** 2017-08-21

**Authors:** William A. Fung, Jiandong Su, Zahi Touma

**Affiliations:** Toronto Western Hospital, Centre for Prognosis Studies in the Rheumatic Diseases, University of Toronto Lupus Clinic, Toronto, ON, Canada

## Abstract

This study aims to elucidate the predictive capabilities of proteinuria, serum creatinine (Cr), and urine RBCs (uRBCs) with respect to long-term renal outcomes in lupus nephritis (LN) in patients followed in clinic.* Methods*. A retrospective analysis was performed on patients with LN. We evaluated the ability of proteinuria, serum Cr, and uRBCs at 12 months to predict good long-term renal outcomes defined as serum Cr ≤ 100 mmol/L and kidney transplant/dialysis-free at the 7th year. Receiver operator characteristic curves were generated for proteinuria, serum Cr, and uRBCs to study their ability to predict good long-term outcomes and to identify their best cut-off. Descriptive statistics studied the pattern of change of proteinuria and serum Cr.* Results*. Proteinuria of 0.6 g/d and Cr of 83 mmol/L performed independently moderately well in predicting good long-term renal outcomes while uRBC was less accurate. Combining serum Cr to proteinuria gave a small increase in positive predictive value with a trade-off in sensitivity. Proteinuria changed within the first year whereas serum Cr changed until the 7th year.* Conclusions*. Both proteinuria and Cr predict good long-term renal outcomes in LN. Proteinuria's ability to change faster at 12 months makes it a favorable endpoint for clinical trials and research studies.

## 1. Introduction

Systemic lupus erythematosus (SLE) is an autoimmune disease with widespread organ involvement. Lupus nephritis (LN) is a common manifestation of SLE associated with significant mortality and morbidity, with a cumulative incidence of 54% [1]. Multiple randomized clinical trials (RCTs) have been performed in recent years investigating different therapeutic modalities for LN [2–4]. These RCTs have used several short-term renal parameters (common endpoints in RCTs are proteinuria, serum creatinine, and urinary sediments (Table 1)) which are potential surrogates that predict long-term response, both renal and extrarenal. The definitions of short-term parameters amongst trials are very heterogeneous wherein each trial has different definitions of partial and complete response, incorporating varying thresholds for proteinuria and serum creatinine (Cr) as well as urinary red blood cells (uRBCs) [2–4]. Certainly the choice of short-term parameters, the variations in the definitions of short-term parameters, and the timing of when they are measured (trial length) can significantly influence the success or failure of a clinical trial [5, 6]. Given that the advent of new therapeutics in trials may depend upon the endpoint used to define success, there needs to be clarification of which endpoints are able to predict good long-term renal outcomes in LN.

Recently, analyses of the Euro-Lupus Nephritis Trial (ELNT) [7] and MAINTAIN Nephritis Trial (MNT) [8] demonstrated that as a short-term parameter, proteinuria at 12 months with a cut-off of 0.8 and 0.7 g/day, respectively, is the best predictor of long-term renal outcome in LN. Touma et al. [9] have demonstrated that the change in proteinuria tends to be slow, with recovery taking up to 2 years. Additionally the long-term follow-up of two lupus nephritis trials (ELNT and MAINTAIN) observed that the 1-year mark was more predictive of long-term renal outcomes compared to 3- and 6-month points (ELNT) [7, 8].

We performed a retrospective analysis on a cohort of SLE patients with LN followed prospectively at the Toronto Lupus Clinic in order to determine the predictive capabilities of Cr, 24-hour urine proteinuria (24H-P), and uRBCs with respect to good long-term renal outcome. Subsequent analyses were also performed looking into the optimal proteinuria cut-off to predict good long-term renal outcome and into the pattern of change in proteinuria and Cr after initial LN episode.

## 2. Methods

### 2.1. Study Cohort

Patients were selected from the prospective longitudinal Toronto Lupus cohort followed from 1970 to April 2016. Patients had SLE (4 or more American College of Rheumatology [ACR] criteria [10, 11] or 3 ACR criteria and a typical biopsy lesion of SLE). Collection, storage, and use of clinical and laboratory data are conducted in accordance with the Declaration of Helsinski and approved by the Research Ethics Board of the University Health Network, Toronto, Canada. Signed informed consent was obtained from all patients.

### 2.2. Patient Assessment

Patients attend the Lupus Clinic at 2- to 6-month intervals irrespective of their disease activity. Patient assessment is performed as per a standard protocol and includes complete history, physical examination, and laboratory evaluation as well as collection of information on medications. For this study we analyzed laboratory results including serum Cr, 24H-P, and uRBCs [12, 13]. The 24H urine sample was collected as follows: patients were instructed to empty their bladder in the morning and discard the urine and then to collect their urine in a container from that point on for 24 hours. At the end of the 24H period, the bladder was to be emptied and that urine saved. Urine specimens are handled and interpreted by laboratory and the appropriate measurements of preservation and shipment of the samples are applied.

### 2.3. Patient Selection

For this study, the first episode of LN, since they joined the clinic, was defined as a one-time 24H-P ≥ 0.5 g/d [14, 15]. Patients with LN and at least 7 years of follow-up thereafter were selected for inclusion. Patients with baseline renal transplant/dialysis or eGFR < 15 mL/min/173 m^2^ were excluded.

### 2.4. Definitions of Renal Parameters

24H-Pand uRBCs are recorded and scored in our database only if they are attributed to SLE activity. The interpretation of the urine analysis is based on physician judgment. eGFR was calculated using the Cockcroft-Gault equation.

### 2.5. Study Design

The renal parameters (24H-P, Cr, and uRBCs) for each patient at 1 year after the diagnosis of LN were studied. Every patient in this study had a baseline (corresponding to the 1st visit in this study) and follow-up 24H-P. Patients were treated with standard of care as determined by the treating rheumatologist.

### 2.6. Study Endpoints

Primary good long-term renal endpoint was defined as Cr ≤ 100 mmol/L at 7th year of LN and no dialysis/renal transplant up to 7th year of LN. Secondary good long-term renal endpoint was eGFR ≥ 60 mL/min/173 m^2^ at 7 years.

### 2.7. Study Analysis

Descriptive statistics were used to describe patient characteristics. Receiver operating characteristic (ROC) curves were generated to examine the predictive power of 24H-P, Cr, and uRBCs (at 12 months) with respect to primary and secondary good long-term renal endpoints. Area under curve (AUC) was analyzed for each baseline endpoint for (a) value at year 1, (b) absolute change from baseline to year 1, and (c) percent change between baseline and year 1. AUC of 0.7–0.9 indicates moderate accuracy, 0.5–0.7 indicates low accuracy, and ≤0.5 is equal to chance [16]. Cut-offs with optimized sensitivity and specificity for each studied endpoint were identified by the Youden index. The performance of the combination of proteinuria with serum Cr at year 1 for predicting good long-term endpoints was as well evaluated. Positive predictive value (PPV) and negative predictive value (NPV) as well as 95% confidence intervals (CI) were determined. Additional descriptive analysis was performed to compare the pattern of change in serum Cr and 24H-P over time following diagnosis of LN and plots were created to visualize the changes. Sensitivity analysis was performed for the subgroup of patients with baseline 24H-P ≥ 1 g/d to determine the optimized 24H-P cut-off for this subgroup.

## 3. Results

### 3.1. Patient Characteristics

In total, 101 patients with at least 7 years of follow-up were included in this study (Table 2). 87% were female, with mean disease duration at LN of 4.34 ± 4.6 years. Mean age at LN was 33.0 ± 10.7 years. The majority of the patients were being treated with glucocorticoids (92.1%) with a smaller percentage (51.5%) receiving antimalarials and immunosuppressives (59.4%). At baseline, mean SLEDAI-2K was 13.3 ± 7.1, and SDI was 0.44 ± 0.95. Of those who had renal biopsies (*n* = 78), the majority (*n* = 48) were World Health Organization (WHO)/International Society of Nephrology (ISN) class IV or V. 40 patients (39.6%) were being treated with ACE inhibitor or ARB by one year after LN diagnosis.

### 3.2. Laboratory Results

The baseline median 24H-P was 0.6 g/d with mean 1.17 ± 1.59 g/d. Baseline median serum Cr was 75.0 (interquartile range: 62.0–89.0) mmol/L, while at 7 years it was 72.0 (62.0–95.0) mmol/L. One patient ended up requiring dialysis/renal transplant, whereas 19 patients (18.8%) had an eGFR < 60 at 7 years.

### 3.3. ROC Curves Analyses for Proteinuria, Serum Cr and uRBCs

#### 3.3.1. Proteinuria

**Figure 1:**
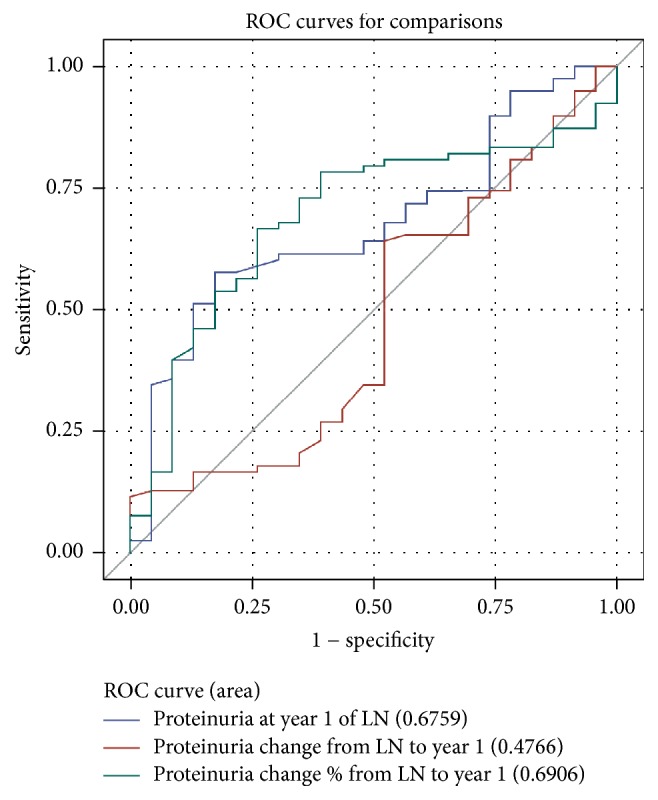
ROC curves for predictive value of proteinuria at 1 year for primary good long-term renal endpoints. Proteinuria at 1 year and percentage change from baseline to year 1 demonstrate departure from the line of unity, whereas absolute proteinuria from baseline to year 1 does not. In all ROC curves sensitivity is plotted on the *y*-axis against 1 − specificity on the *x*-axis.

Overall, the proteinuria value at 1 year (AUC 0.68) and proteinuria percent change from baseline to year 1 (AUC 0.69) predicted good long-term renal endpoints while absolute change from baseline to year 1 did not (AUC 0.47) (Figure 1). For the primary endpoint (Cr ≤ 100 mmol/L at 7th year of LN and no dialysis/renal transplant up to 7th year of LN), the optimized cut-off of proteinuria was 0.57 g/d. For the secondary good long-term renal endpoint (eGFR ≥ 60 mL/min/173 m^2^ at year 7) the optimized proteinuria cut-off was 0.44 g/d with AUC 0.65 for proteinuria value at 1 year and AUC 0.70 for proteinuria percent change from baseline to year 1. The sensitivity and specificity were 0.58/0.83 and 0.50/0.89 for the endpoints of Cr ≤ 100 mmol/L and eGFR ≥ 60 mL/min/173 m^2^ at year 7, respectively. The optimized cut-off for percent change in proteinuria from baseline to year 1 for the primary endpoint was 54.5% (sensitivity 67 and specificity 74).

**Figure 2:**
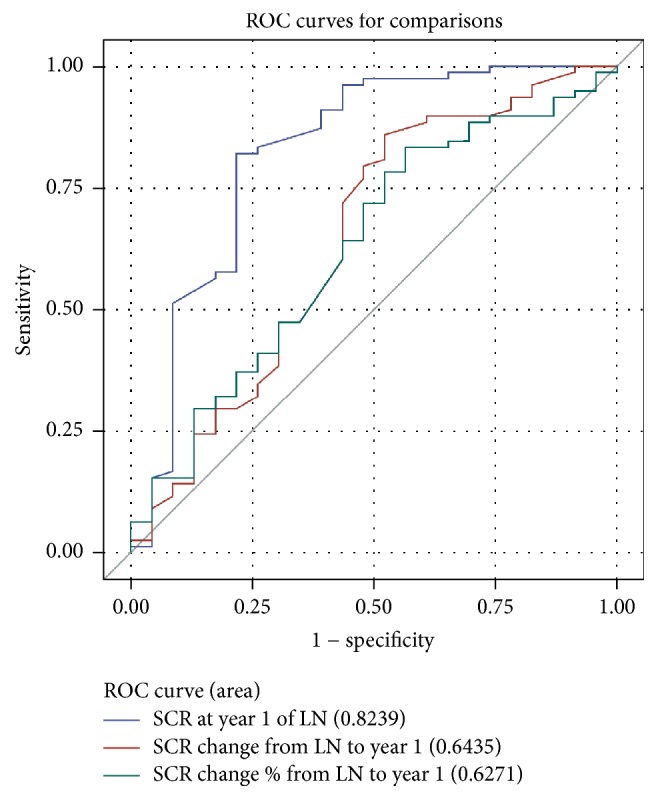
ROC curves for predictive value of serum Cr at 1 year for primary good long-term renal endpoints. All the ROC curves for serum Cr demonstrate departure from unity with the best AUC of 0.82 for serum Cr value at year 1. Serum Cr 1 year and percentage change from baseline to year 1 demonstrate departure from the line of unity, whereas absolute proteinuria from baseline to year 1 does not.

Conversely, ROC curves for serum Cr all demonstrated departure from unity, with the best being absolute baseline Cr, with an AUC of 0.82 (Figure 2). The optimal cut-off of Cr for the primary endpoint was 83 mmol/L, with a sensitivity of 0.82 and specificity of 0.78. For the secondary endpoint (eGFR ≥ 60), the optimal Cr cut-off was 111 mmol/L, with a sensitivity of 0.97 and specificity of 0.63.

**Figure 3:**
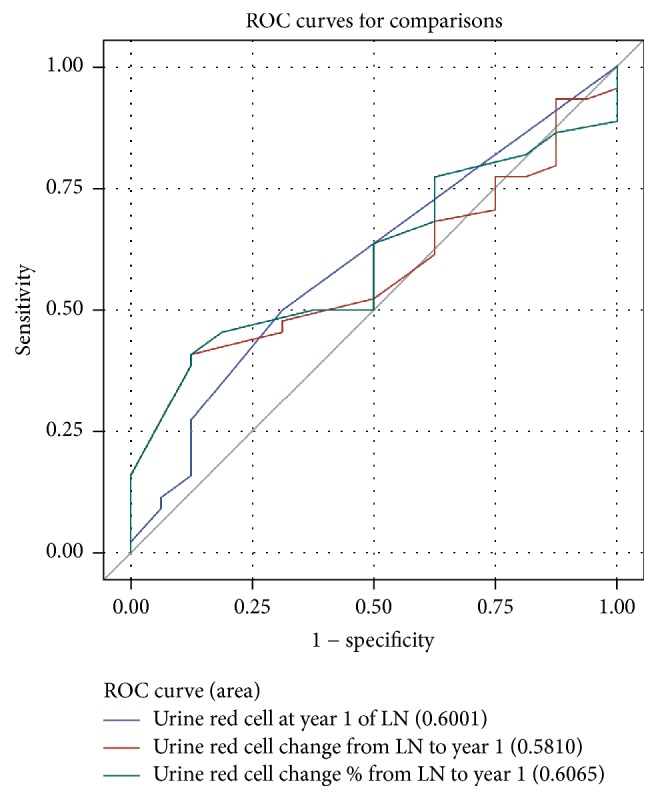
ROC curves for predictive value of uRBCs at 1 year for primary good long-term renal endpoints. uRBCs at year 1 and uRBCs percentage change from baseline to year 1 demonstrate departure from the line of unity.

All the ROC curves for uRBCs at year 1 indicate low accuracy with AUC 0.60 for uRBCs at year 1 and uRBCs percentage change from baseline to year 1 (Figure 3).

**Figure 4:**
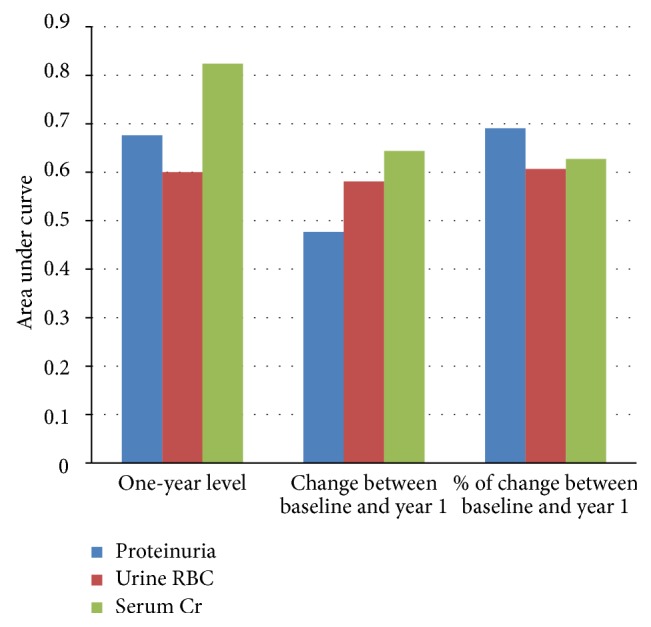
Comparison of the area under the curve (AUC) for proteinuria, uRBC, and serum Cr at 1 year quantified as the one year level, the change from baseline visit, and the percentage change from baseline.

Figure 4 illustrates the AUC values for proteinuria, serum Cr, and uRBCs measured as the value at year 1, absolute change from baseline to year 1, and percent change between baseline and year. Overall, the AUCs for all 3 short-term parameters, proteinuria, serum Cr, and uRBCs, were best for the value of year 1.

The performance of 24H-P and Cr individually and in combination is summarized in Table 3. Combining proteinuria and serum Cr causes a small increase in the PPV, but with the trade-off of decreased sensitivity.

### 3.4. Pattern of Changes within 7 Years after LN

**Figure 5:**
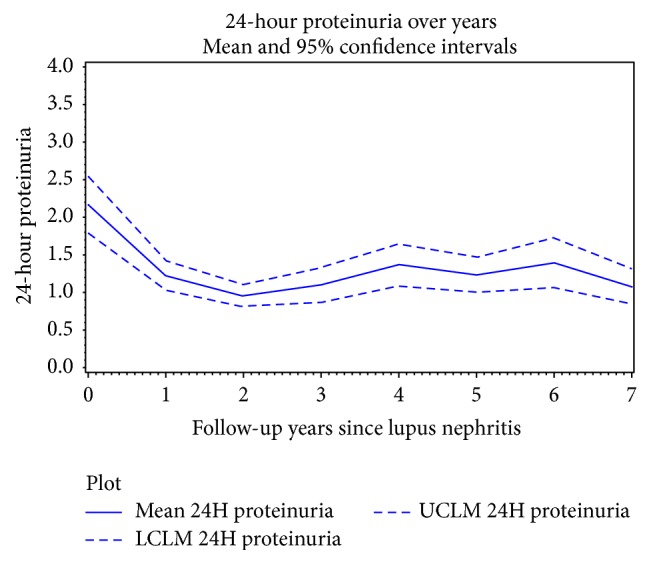
Change in proteinuria during follow-up. After initiation of therapy at LN diagnosis, there is rapid decrease in 24H-P that remains relatively stable thereafter. LCLM lower confidence limit. UCLM upper confidence limit.

**Figure 6:**
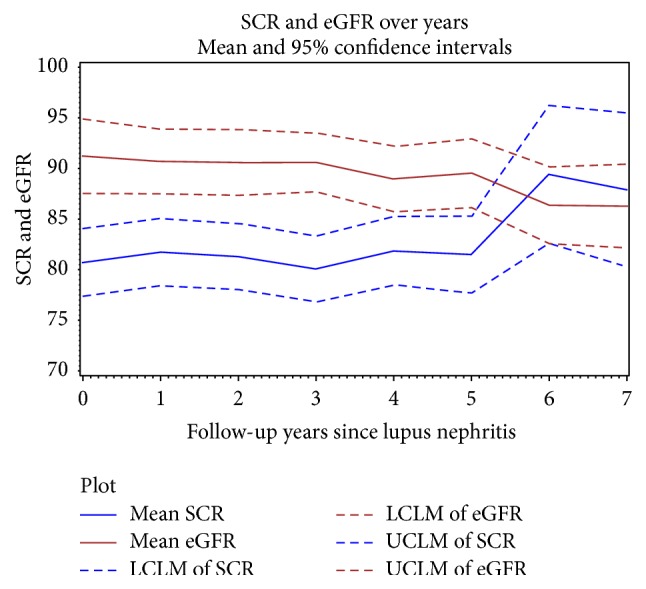
Change in serum Cr and eGFR during follow-up. After initiation of therapy at LN diagnosis, serum Cr and eGFR remain relatively unchanged until year 6.

Examination of the change in 24H-P and serum Cr in response to therapeutic intervention after the diagnosis of LN demonstrated that the pattern of change for these parameters is different. Change in 24H-P was largest within the first year after LN diagnosis (Figure 5), whereas for serum Cr, the largest change occurred later, between the 5th and 6th year after LN diagnosis (Figure 6). 24H-P therefore changes rapidly after the institution of therapy whereas the response of Cr is delayed.

### 3.5. Sensitivity Analysis

**Figure 7:**
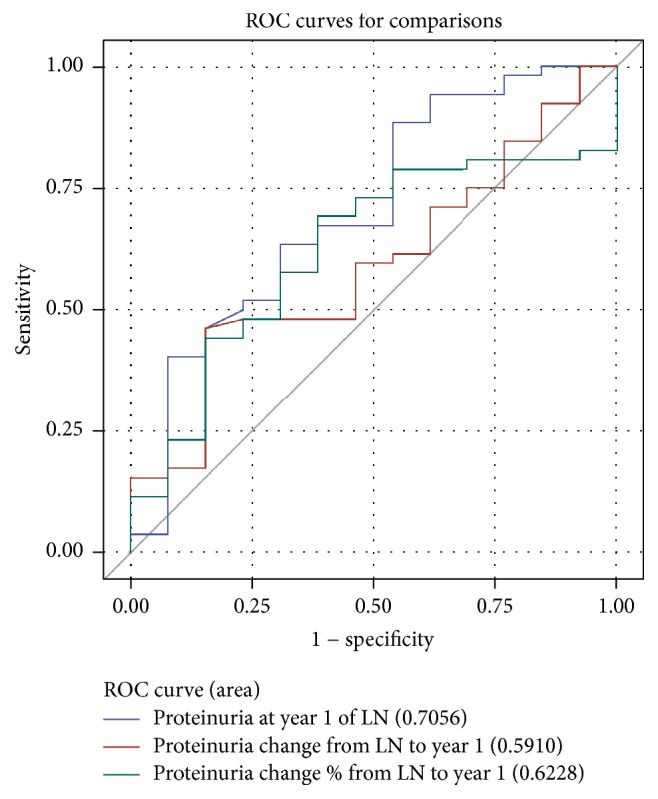
ROC curves for predictive power of 24H-P at 1 year for primary renal outcomes in subgroup with baseline 24H-P ≥ 1 g/d.

65 patients with baseline 24H-P ≥ 1 g/d were identified. As with the original analysis, the 24H-P at 1 year was a moderate predictor of good long-term renal outcomes (AUC 0.71) and fared better than both the absolute and percentage changes at 1 year (Figure 7). The optimal cut-off by Youden index was 24H-P of 0.95 at 1 year for this subgroup (Table 4). The proteinuria cut-off of 0.6 g/d showed a sensitivity and specificity of 51 and 77%, respectively.

## 4. Discussion

The majority of recent RCTs in LN therapeutics have resulted in nonsignificant differences compared to control groups, which at least partly has been affected by definitions of endpoints along with other factors related to study design [5, 6]. In addition, there is no agreement on the definitions of endpoint for LN RCTs (Table 1). Although a renal activity score and renal response index were developed for this purpose, their uses in RCTs have been seldom [26]. Therefore there is an emerging need to better define which of these measurements reflect good long-term outcomes.

Our analysis demonstrates that 24H-P at 1 year post-LN is a fair predictor of good long-term renal outcome, with an AUC of 0.68 for the outcome of the combination of serum Cr ≤ 100 mmol/L and no dialysis/renal transplant and 0.65 for the outcome of eGFR ≥ 60 mL/min/173 m^2^. Determination of a cut-off for 24H-P was optimized at 0.57 g/d (~0.6 g/d) and 0.44 g/d for primary long-term renal outcomes, which is slightly higher than the current therapeutic target being used in the majority of clinical trials and practice (0.5 g/d) but suggests that the current proteinuria target is reasonable. This is as well slightly different to previous studies that suggested that a proteinuria cut-off of approximately 0.8 g/d would be optimal [7, 8]. One possible explanation is that the population of patients included in these studies had higher baseline 24H-P than our cohort [19, 23]. In support of this, sensitivity analysis performed on the subgroup of patients with baseline 24H-P ≥ 1 g/d demonstrated an optimal 24H-P cut-off of 0.95 g/d (by Youden index) at 1 year, which is close to the cut-offs suggested by these previous studies and which also suggests that the difference in cut-offs is at least partially related to the degree of baseline proteinuria (the cut-off of 0.82 g/d showed a sensitivity and specificity of 56 and 69%, resp.) (Table 4). The importance of our results is that they are derived from patients followed in clinic, reflecting real-life, compared to the results of the previous 2 studies on patients followed in clinical trials [7, 8].

Although uRBCs provide low accuracy in predicting long-term renal outcomes with AUC of 0.60 its utility as an endpoint in clinical trials should be determined carefully. Recently, Dall'Era et al. [7] and Tamirou et al. [8] highlighted the difficulties associated with the quantification of uRBCs in clinical trials. Dall'Era et al. stated that the measurement of uRBCs often is reported as a range as opposed to a continuous value which prevents the calculation of a change from baseline [7]. Tamirou et al. explained how crucial it is to consider several factors that could hinder the reliability and precision of uRBCs measurement; some of these factors are related to the time of urine collection, preservation of urine, and standardization in the methods of measurement of uRBCs [8]. Thus, all these factors need to be addressed before considering uRBCs as an endpoint in trials. Wofsy et al. showed that a definition of complete response that does not include urinary sediment (amongst other differences) would generate higher complete response rates using the same data set [5].

Serum Cr at 1 year was a moderately good predictor of good long-term renal outcome, with an AUC of 0.82 for primary outcome and 0.85 for the secondary outcome. Furthermore, our results also demonstrated that serum Cr and eGFR are fairly stable until 6 years after LN onset. This suggests that serum Cr at 1 year is a good predictor that it in fact does provide information about the future trajectory of a patient's renal function. The time frame in RCTs of LN is often 12 months and in this study we demonstrated that the largest change in proteinuria was observed in the first year after LN onset. Given this, although serum Cr is overall a better predictor for long-term renal outcomes, proteinuria is more appropriate as a marker in clinical trials.

Interestingly, analysis of the combined proteinuria and serum Cr cut-offs demonstrated comparable PPV and NPV to serum Cr alone but sacrificed total sensitivity. Compared to proteinuria alone there was an improvement in the specificity, whereas sensitivity, PPV, and NPV are comparable given the CI overlap. This suggests that while 24H-P may be more appropriate as a biomarker due to its sensitivity to short-term change, there may be value in combining it with Cr.

Limitations of our study include the relatively small sample size and retrospective nature of the analysis. Conversely, the strengths of this study include the long-term data available on our cohort of different ethnicities as well as the standardized protocol under which this data is collected.

Overall, our study demonstrates that proteinuria value at 1 year with a cut-off of 0.6 g/day at 1 year predicts good long-term renal outcomes. Thus, proteinuria and possibly serum Cr can serve as good endpoints in LN clinical trials and research studies. Both the low accuracy of uRBCs in predicting good long-term renal outcomes and the lack of precision in its measurement render it a less appealing endpoint for clinic trials.

## Figures and Tables

**Table 1:** Recent trials of therapeutics in LN and their endpoints.

Trial	Agent	Primary endpoints	Time of evaluation
LUNAR [17]	Rituximab	Serum Cr normal or ≤115% of baseline, inactive urinary sediment, and protein-creatinine ratio (PCR) < 0.5	52 weeks

BELONG [18]	Ocrelizumab	Cr ≤ 25% increase from baseline and PCR < 0.5	48 weeks

ELNT [19]	Cyclophosphamide	Treatment failure: Cr ≥ 1.3 mg/dl or Cr improvement < 50% or persistence of nephrotic syndrome Flare not responding to 1-month increased glucocorticoids Doubling of Cr over lowest value at any time	6 months

Mycophenolate mofetil versus cyclophosphamide for induction treatment of lupus nephritis [20]	Mycophenolate mofetil	PCR < 3 if baseline is nephrotic, or improvement ≥ 50% if subnephrotic	24 weeks

Mycophenolate versus azathioprine as maintenance therapy for lupus nephritis [21]	Mycophenolate mofetil	Time until 1st event: death, ESRD, sustained Cr doubling, renal flare, or need for rescue therapy Proteinuric flare: doubling of PCR Nephritic flare: increase of 25% or more in lowest Cr, plus at least one of doubling of urinary protein clearance, new or increased hematuria, or cellular casts	36 months

Efficacy and safety of abatacept in lupus nephritis: a twelve-month, randomized, double-blind study [22]	Abatacept	eGFR ≥ 90% of baseline, PCR < 0.25 gm/gm, inactive urinary sediment	12 months

MAINTAIN [23]	Mycophenolate mofetil	Time to renal flare: recurrence/development of nephrotic syndrome, ≥33% increase in Cr attributed to SLE, or 3-fold increase of 24H-P within 3-month period accompanied by uRBCs, and >33% reduction of serum C3	48 months

Mycophenolate mofetil or intravenous cyclophosphamide for lupus nephritis [24]	Mycophenolate mofetil	Return to within 10% of normal values of Cr, 24H-P, and uRBCs	24 weeks

ACCESS [25]	Abatacept	All of PCR < 0.5, Cr ≤ 1.2 mg/dL or ≤125% of baseline, and adherence to prednisone taper	24 weeks

**Table 2:** Patient demographics at the diagnosis of LN.

Demographics	
Female	87 (86.1%)
Ethnicity	
Caucasian	63 (62.4%)
Black	18 (17.8%)
Asian	12 (11.9%)
Others	8 (7.9%)
Age at SLE diagnosis (years)	28.64 ± 10.89
Age at LN (years)	32.99 ± 10.66
Disease duration at LN (years)	4.36 ± 4.60
*Disease scores*	
SLEDAI-2K at LN diagnosis	13.29 ± 7.14
SDI at LN diagnosis	0.44 ± 0.95
*Treatment*	
Treated with glucocorticoids at LN	93 (92.1%)
Treated with antimalarials at LN	52 (51.5%)
Treated with immunosuppressives at LN	60 (59.4%)
Treated with ACE inhibitor/ARB	40 (39.6%)
Laboratory tests at LN diagnosis	
24H-P (g/d) median (interquartile range)	1.5 (0.9–2.9)
24H-P (g/d) mean at baseline	2.36 ± 2.31
24H-P (g/d) mean at year 1	1.17 ± 1.59
uRBCs (hpf) median (interquartile range)	5.0 (0.0–10.0)
Cr (mmol/L) median (interquartile range)	72.0 (64.0–89.0)

**Table 3:** Performance of proteinuria and serum Cr cut-offs at year 1 to predict good long-term renal outcomes at 7 years.

Measures	Sensitivity (95% CI)	Specificity (95% CI)	PPV (95% CI)	NPV (95% CI)
24H-P < 0.57 g/d	0.58 (0.47–0.69)	0.83 (0.67–0.98)	0.92 (0.84–0.99)	0.36 (0.23–0.50)
Cr < 83 mmol/L	0.82 (0.73–0.91)	0.78 (0.61–0.95)	0.93 (0.87–0.99)	0.56 (0.39–0.73)
24H-P < 0.57 g/d and Cr < 83 mmol/L	0.51 (0.40–0.62)	0.96 (0.87–1.00)	0.98 (0.93–1.00)	0.37 (0.24–0.49)

*Note*. PPV: positive predictive value; NPV: negative predictive value; CI: confidence intervals.

**Table 4:** Performance of proteinuria cut-offs at 1 year to predict good long-term renal outcomes in subgroup of patients with baseline proteinuria ≥ 1 g/d.

24H-P at 1 year	Sensitivity	Specificity	PPV	NPV
0.61	0.51	0.77	0.90	0.29
0.66	0.52	0.70	0.87	0.26
0.82	0.56	0.69	0.88	0.28
0.90	0.60	0.69	0.89	0.30
0.94	0.62	0.69	0.89	0.31
0.95^*∗*^	0.63	0.69	0.89	0.32
1.01	0.63	0.62	0.86	0.30

PPV = positive predictive value; NPV = negative predictive value. ^*∗*^Best cut-off by Youden index.

## Additional Points

*Significances*. (i) Proteinuria value at 1 year with a cut-off of 0.6 g/day at 12 months is a good predictor for good long-term renal outcomes. (ii) This study on patients followed in clinic validates the findings derived from clinical trials and confirms that proteinuria value at 1 year is the best predictor for long-term renal outcomes. (iii) Although serum Cr of 83 mmol/L predicted good long-term renal outcomes, the ability of proteinuria to change significantly at 12 months makes it the favorable endpoint.
